# Comparison of frequency of asymptomatic microhematuria in patients with stage 2-4 versus stage 0-1 pelvic organ prolapse

**DOI:** 10.12669/pjms.313.6934

**Published:** 2015

**Authors:** Emrah Töz, Sefa Kurt, Mehmet Tunç Canda, Çağdaş Şahin, Ibrahim Uyar

**Affiliations:** 1 Emrah Töz, Tepecik Training and Research Hospital, Department of Obstetrics and Gynecology, Izmir 35170, Turkey; 2 Sefa Kurt, Department of Obstetrics and Gynecology, Izmir Dokuz Eylül University, Izmir, Turkey; 3 Mehmet Tunç Canda, Department of Obstetrics and Gynecology, Kent Hospital, Izmir 35580, Turkey; 4 Çağdaş Şahin, Tepecik Training and Research Hospital, Department of Obstetrics and Gynecology, Izmir 35170, Turkey; 5 Ibrahim Uyar, Tepecik Training and Research Hospital, Department of Obstetrics and Gynecology, Izmir 35170, Turkey

**Keywords:** Asymptomatic microhematuria, Pelvic organ prolapse

## Abstract

**Objective:**

To compare the frequency of asymptomatic microhematuria (AMH) in patients with stage 2-4 and stage 0-1 pelvic organ prolapse (POP).

**Methods:**

The hospital database was searched for women diagnosed with pelvic floor disorders and all medical records were reviewed retrospectively for the presence of AMH. An additional search was conducted for women with other benign gynecological conditions such as myoma uteri, endometrial hyperplasia or adnexal masses without evidence of pelvic organ prolapse (control group). The control group was created using 1:1 matching for age and menopausal status. The frequency of AMH in these patients were compared. The degree of hematuria was categorized as reported by the laboratory as 3 to 25 (low grade hematuria), 26 to 50 (intermediate grade hematuria) and 51 or more (high grade hematuria) red blood cell/high powered field.

**Results:**

AMH is statistically significant more often seen in study group than in control group (p:0.016). In the prolapse group 20 women (13.7%) had AMH compared with 9 (6.2%) in the control group. All of 29 patients with AMH had low grade hematuria defined as < 25 red blood cell/high powered field. Patients were followed up for 22 ± 7 (12 to 33) months. No bladder cancer and no cancer of the upper urinary tract has been detected in these 29 patients with AMH during follow-up.

**Conclusions:**

Women with stage 2-4 POP are more likely to be diagnosed with AMH than those with stage 0-1 prolapse.

## INTRODUCTION

The definition of hematuria is the presence of red blood cells in the urine. When visible it is termed gross hematuria and when detected by microscopic examination of the urinary sediment it is termed microscopic hematuria or microhematuria. Microhematuria may also be either symptomatic (unilateral flank pain, lower irritative voiding symptoms, recurrent urinary tract infections despite appropriate use of antibiotics, etc) or asymptomatic. Dipsticks have a sensitivity of 95% and a specificity of 75% but positive results should be confirmed with a microscopic examination of the urine because free hemoglobin, myoglobin and antiseptic solutions like povidone-iodine, will also give positive readings.^1^ With microscopic examination of urine, asymptomatic microhematuria (AMH) is defined by the presence of three or more red blood cells (RBC) per high powered field (HPF) on one properly collected urinary specimen in the absence of an obvious benign cause.^1^

The adult population prevalence of microhematuria ranges from 2.4% to 31.1% depending on age, gender, frequency of testing, threshold used to define microhematuria and study group characteristics such as the presence of risk factors.^2^-^5^ According to the guidelines, once the microhematuria diagnosis is made, further costly and burdensome tests (cystoscopy, multi-phasic computed tomography urography etc.) should be performed after the benign causes have been ruled out. But those guidelines were based on studies that include both genders in their analyses. The research on women has been limited and the results of women with pelvic organ prolapse (POP) are controversial. Previous research suggested that prolapse is not related to the presence or absence of AMH but a new study suggests that AMH rates are higher in women with pelvic prolapsed.^6^,^7^ They hypothesized that women with POP have an increased frequency of AMH because of the mucosal trauma incited by the prolapse.^6^,^7^

This study aimed to compare the frequency of asymptomatic microhematuria (AMH) in patients with stage 2-4 and stage 0-1 POP. We hypothesis that women with stage 2-4 POP have an increased frequency of AMH. Therefore, women with stage 2-4 POP and with stage 0-1 POP were included. The frequency of AMH in these patients were compared.

## METHODS

This retrospective follow up study was conducted in the Obstetrics and Gynecology Department at Tepecik Training and Research Hospital, Izmir, Turkey. The study was approved by the local ethics committee and institutional review board. The hospital database was searched for women diagnosed with at least one of the following pelvic floor disorders: descensus uteri, cystocele or cysto-rectocele, and with or without symptomatic urinary incontinence (study group) between January 2011 and January 2012. The grade of urogenital prolapse was classified according to the pelvic organ prolapse quantification system (POP-Q). Patients with stage 2-4 prolapse were included in the study group. An additional search was conducted for women with other benign gynecological conditions such as myoma uteri, endometrial hyperplasia or adnexal masses without evidence of pelvic organ prolapsed (control group cohort).145 patients with stage 2-4 prolapse were eligible for the study. The final control group was created from control group cohort using 1:1 matching for age, parity and menopausal status with SAS version 9.1 (SAS Institute Inc., Cary, NC, USA). Exclusion criteria were: no recorded urine dipstick, irritating voiding symptoms, diagnosis of interstitial cystitis, anamnesis of trauma or vigorous exercise, macroscobic hematuria, use of hormone replacement therapy, urinary tract infection or viral illness, menses at time of visit and pregnancy.

All medical records were reviewed for the presence of AMH on urine dipstick. If AMH was present, a urine microscopy was required to be present to document AMH. Demographic characteristics including age, parity, mode of delivery, smoking and menopausal status, body mass index (BMI) and presence/absence of hypertension and diabetes were recorded. Both groups were compared in terms of the presence of AMH.

For microscopic examination, 10 ml freshly voided clean-catch mid-stream urine sample was centrifuged at 2,000 revolutions per minute for 10 minutes. Then sediments resuspended in 0.3 mL saline and placed on a microscopic slide.10 microscopic fields were examined under 400x magnification. The count was recorded as per high power field for red blood cells.

According to previous study,^7^ a minimum sample size of 106 for each group would achieve 80% statistical power for detection of a difference for the presence of AMH, using p<0.05 to indicate significance. In the light of this data, our study’s statistical power was sufficient to reach a significant conclusion. Chi-squared tests were used to analyse qualitative variances. Multivariate linear regression analyses were conducted to evaluate the association between POP and AMH after adjusting for covariates (age, BMI, hypertension, diabetes, smoking and menopausal status). Descriptive statistics were used to constitute demographic characteristics. A threshold of p<0.05 was considered to indicate statistical significance. Data were analysed using Statistical Package for the Social Sciences version 18.0 (SPSS Inc., Chicago IL, USA).

## RESULTS

The mean age of the 290 participants was 54.91 ± 4.85 years. Demographic and clinical characteristics of the two groups are summarised in Table-I. Age, BMI, parity, mode of delivery, menopausal status, smoking status, and presence/absence of hypertension and diabetes did not differ significantly between the two groups.

**Table-I:** Clinical and demographic characteristics of the study and control groups.

Characteristics	Study group (n = 145)	Control group (n = 145)	p-value
Age (years)	54.60±4.73 (48-63)	55.24 ±5.36 (48-64)	0.222
Body mass index (kg/m2)	29.36±3.41(23-37)	28.86± 3.31 (24-36)	0.479
Parity	3.34±1.28 (2-6)	3.44±1.32 (2-7)	0.142
Vaginal birth	129(89%)	133 (92%)	0.174
Caesarean section	16(11%)	12 (8%)	0.242
Postmenopausal status	84(58%)	81(56%)	0.084
Smoking status	4(3%)	7 (5%)	0.070
Hypertension	39(27%)	33 (23%)	0.061
Diabetes mellitus	9(6%)	12 (8%)	0.059
AMH *	20(13.7%)	9 (6.2%)	0.016

Data are shown as mean Standard deviation (range) or n (%),

* Asymptomatic microscopic hematuria.

The overall prevalence of AMH was % 10 (29/290) in a cohort of 290 patients. AMH was statistically significant more often seen in study group than in control group (p:0.016). In the study group 20 women (13.7%) had AMH compared with 9 (6.2%) in the control group. Multivariate analyses controlling for age, BMI, menopausal status, smoking status and co-morbidities revealed that women with stage 2-4 pelvic prolapse had increased risk of AMH (odds ratio 2.41, 95% confidence interval 1.06 – 5.50) compared with women with stage 0-1 POP.

After the initial analysis, urinalysis results were stratified as 0 to 2, 3 to 10, 11 to 25, 26 to 50, and more than 50 RBC/HPF. Patients with AMH (three or more RBC/HPF) were assessed for degree of hematuria. The degree of hematuria was categorized as reported by the laboratory as 3 to 25 (low grade hematuria), 26 to 50 (intermediate grade hematuria) and 51 or more (high grade hematuria) red blood cell/high powered field. All of 29 patients with AMH had low grade hematuria, defined as < 25 RBC/HPF (Table-II).

**Table-II:** The degree of hematuria in all study cohort.

RBC/HPF	Study group (n=145)	Control group (n=145)
0-2	125 (86%)	136 (94%)
3-10	16 (11%)	8 (5%)
11-25	4 (3%)	1 (1%)

RBC: red blood cells, HPF: high powered field.

After the appropriate evaluation by urologist, patients were followed up for 22 ± 7 (12 to 33) months. No bladder cancer and no cancer of the upper urinary tract have been detected in these 29 patients with AMH during follow-up.

## DISCUSSION

This study found that women with stage 2-4 pelvic prolapse are more likely to be diagnosed with AMH than those with stage 0-1 prolapse. The prevalence of AMH in this study [10% (29/290)] was lower than in some previous studies, which found rates of 16%^8^ and 21%.^9^ This is attributed to patients’ characteristics. Differences in the age and sex of the populations screened account for this difference. Our study did not include patients who were at a high risk for urologic disease such as older men.

Mucosal trauma caused by the prolapse may cause hematuria. This is in contrast to previous research suggesting that stage of prolapse is not related to the presence or absence of AMH. However, this is consistent with conclusions that AMH rates are higher in women with prolapse.^5^,^6^ We also found that women with stage 2-4 pelvic prolapse are more likely to be diagnosed with AMH.

Blood in the urine can originate at any point along the urinary tract and may represent serious underlying disease. American Urological Association (AUA) recommends multi-phasic computed tomography urography and cystoscopy for evaluation of AMH after one positive urinalysis. On the other hand, the frequency of serious urological disease in patients with AMH is low (malignant origins range from 0.5% to 5.0%).^1^,^10^,^11^ We also found no bladder cancer and no cancer of the upper urinary tract in 29 patients with AMH during follow-up.

Thus, it is recognized that by following the current guidelines, many patients will undergo costly diagnostic procedures and exposed to radiation by multi-phasic computed tomography without any benefit. To avoid this, some effort has been made in developing a method that identify patients who should undergo further diagnostic evaluation. Using the risk factors such as history of gross hematuria, age, sex, smoking history and high grade hematuria, a Hematuria risk Index was created to predict cancer risk.^12^ According to this Hematuria risk Index, low-grade hematuria (<25 RBC/HPF) was not a reliable indicator of the presence of urologic malignant tumors, the overall 3-year incidence of urinary tract cancer was only 0.43%.^13^ In this study we found that women with prolapse are more likely to be diagnosed with AMH but hematuria is low grade (<25 RBC/HPF) in all patients, which doesn’t mean high risk for urologic malignant tumors.

Strengths of our analysis include a large sample size and a comparable number of women both with stage 2-4 and stage 0-1 prolapse. And also long follow-up period after the initial evaluation for AMH enhanced the validity of the study.

The results of this study show that women with stage 2-4 POP are more likely to be diagnosed with AMH than those with stage 0-1 prolapse. In future this finding should be integrated in the planning of risk indexes. But these datas are not enough to suggest a new policy to evaluate hematuria in women. Further studies are needed to report specific guidelines for women and reduce the number of unnecessary and costly diagnostic evaluations for AMH.

## References

[1] Davis R, Jones JS, Barocas DA, Castle EP, Lang EK, Leveillee RJ (2012). Diagnosis, evaluation and follow-up of asymptomatic microhematuria (AMH) in adults: AUA guideline. J Urol.

[2] Huussen J, Koene RA, Meuleman EJ, Hilbrands LB (2006). Diagnostic approach in patients with asymptomatic haematuria: efficient or not?. Int J Clin Pract.

[3] Steiner H, Bergmeister M, Verdorfer I, Granig T, Mikuz G, Bartsch G (2008). Early results of bladder-cancer screening in a high-risk population of heavy smokers. BJU Int.

[4] Hedelin H, Jonsson K, Salomonsson K, Boman H (2006). Screening for bladder tumours in men aged 60-70 years with a bladder tumour marker (UBC) and dipstick-detected haematuria using both white-light and fluorescence cystoscopy. Scand J Urol Nephrol.

[5] Brown MA, Holt JL, Mangos GJ, Murray N, Curtis J, Homer C (2005). Microscopic hematuria in pregnancy: Relevance to pregnancy outcome. Am J Kidney Dis.

[6] Shalom DF, Lin SN, St Louis S, Lind LR, Winkler HA (2011). The prevalence of microscopic hematuria in women with pelvic organ prolapse. Female Pelvic Med Reconstr Surg.

[7] Hema DB, David MO, Adam CS (2014). Do Patients With Pelvic Organ Prolapse Have an Increased Frequency of Asymptomatic Microscopic Hematuria?. J Urol.

[8] Woolhandler S, Pels RJ, Bor DH, Himmelstein DU, Lawrence RS (1989). Dipstick urinalysis screening of asymptomatic adults for urinary tract disorders.I. Hematuria and proteinuria. JAMA.

[9] Pillalamarri N, Shalom D, Sanidad S, Akerman M, Lind L, Winkler H (2015). The prevalence of microscopic hematuria in a cohort of women with pelvic organ prolapse. Int Urogynecol J.

[10] Mohr DN, Offord KP, Owen RA, Melton LJ (1986). Asymptomatic microhematuria and urologic disease: a population-based study. JAMA.

[11] Cohen RA, Brown RS (2003). Microscopic hematuria. N Engl J Med.

[12] Loo RK, Lieberman SF, Slezak JM, Landa HM, Mariani AJ, Nicolaisen G (2013). Stratifying risk of urinary malignant tumors in patients with asymptomatic microscopic hematuria. Mayo Clinic Proc.

[13] Jung H, Gleason JM, Loo RK, Patel HS, Slezak JM, Jacobsen SJ (2011). Association of hematuria on microscopic urinalysis and risk of urinary tract cancer. J Urol.

